# Alternative Hospital Gift Bags and Breastfeeding Exclusivity

**DOI:** 10.5402/2013/560810

**Published:** 2013-06-27

**Authors:** Yeon Bai, Shahla M. Wunderlich, Rickie Kashdan

**Affiliations:** ^1^Department of Health and Nutrition Sciences, Montclair State University, 1 Normal Avenue, Montclair, NJ 07043, USA; ^2^Jersey Shore University Medical Center, Family Health Center-Prenatal, 71 Davis Avenue, Neptune, NJ 07753, USA

## Abstract

The type of gift bags given to new mothers at the time of discharge from the hospital can influence their confidence in breastfeeding. Most hospitals in the US continue to distribute commercial gift bags containing formula samples despite the reported negative influence of commercial bags on the duration of breastfeeding. This study compared breastfeeding outcomes in women receiving three different kinds of gift bags at discharge. A prospective intervention study was conducted during 2009-2010 in New Jersey. Three breastfeeding cohorts were recruited and assigned to three groups: COMMERCIAL received discharge bags containing formula samples, BF-INFO received breastfeeding information and supplies, and PUMP received breastfeeding information/supplies plus a manual breast pump. Follow-up contacts were at 2, 4, and 12 postpartum weeks to determine breastfeeding outcome. The mean durations of exclusive (EBF) and partial breastfeeding were compared between groups using ANOVA. A total of 386 participants completed the study. The mean EBF duration (weeks) in the PUMP (*n* = 138, 8.28 ± 4.86) and BF-INFO (*n* = 121, 7.87 ± 4.63) were significantly longer (*P* < 0.01) than COMMERCIAL (*n* = 127, 6.12 ± 4.49). The rate of EBF through 12 weeks in PUMP was most consistent. The mean duration of partial breastfeeding showed similar results: significantly longer in PUMP and BF-INFO than COMMERCIAL (*P* < 0.01).

## 1. Introduction

Breast milk provides an abundance of nutrients in bioavailable forms that are crucial for the infant's normal growth and development [1]. Exclusive breastfeeding provides strong protection against lower respiratory tract infections, gastroenteritis, middle ear infections, and childhood obesity [2–5]. Currently only 14.1% of infants in the US are breastfed exclusively through 6 months, below the target rate of 25.5% in the *Healthy People 2020 *objectives [6]. 

Breastfeeding mothers may encounter cultural and commercial barriers that make it difficult for them to sustain exclusive breastfeeding for the recommended duration [7]. Due to escalating acceptance of infant formula use by doctors and hospitals, breastfeeding can become something people feel they can opt in or out of and may lose its place as an essential part of infant development [8, 9]. 

Following the birth of a baby, information given to the mother can influence her confidence and adaptation to breastfeeding. Hospital practices that avoid formula supplementation and encourage early maternal contact with the newborn (e.g., holding baby skin-to-skin right after birth) and rooming-in support breastfeeding. However, by distributing commercial gift bags containing formula samples, hospitals are inadvertently endorsing formula feeding, an action that has been associated with reducing exclusive breastfeeding rates [7, 10]. This subtle endorsement appears to increase early formula supplementation leading to untimely termination of breastfeeding, particularly with mothers who have unclear or short breastfeeding goals [11, 12]. 

In earlier findings, researchers [13] recommended that materials consistent with the World Health Organization Code (“no free samples to mothers,” and “no promotion products in Health Care Facilities including the distribution of free or low-cost supplies”) replace commercial discharge materials for breastfeeding women. Some states have made active efforts to remove formula samples from hospitals. For example, the launch of a major breastfeeding initiative in 2006 by the New York City Department of Health and Mental Hygiene led to the elimination of formula sample packs from all 11 public hospitals operated by the New York City Health and Hospitals Corporation [14]. 

However, most hospitals in the US continue to distribute commercial discharge bags packaged as smart diaper bags containing various coupons, advertisements, baby products, and infant formula samples. In 2008, Merewood et al. [15] examined 21 eastern states and Washington DC for their gift pack distribution practice. They found that formula sample distribution was still a common practice in eastern states; that is, 94% of hospitals distributed formula sample packs to new mothers at discharge, ranging from 70% to 100%. New Jersey was one of four states in which all maternity hospitals distributed discharge bags containing sample formulas. Merewood et al. concluded that “elimination of sample packs was ongoing … increasing number of hospitals was discontinuing the practice, with regional differences; for example, distribution was least prevalent in New England and was most prevalent in DC and neighboring states” [15]. 

Though a number of studies have examined the effect of commercial discharge bags containing formula samples on the duration of breastfeeding, studies that examine the impact of varying content of hospital discharge bags on breastfeeding duration are few and outdated [16–19]. In order to promote exclusive breastfeeding, a closer examination of the usage of hospital discharge bags is warranted. 

## 2. Materials and Methods

The purpose of this study was to compare the effects of innovative discharge gift bags on the exclusivity and duration of breastfeeding compared to commercial discharge bags. The specific research hypotheses were that (1) the content of discharge gift bags will have an impact on the exclusivity and duration of breastfeeding, and (2) mothers who receive the innovative discharge gift bags will likely to breastfeed longer than mothers who receive traditional commercial discharge bags.

The commercial gift bags (COMMERCIAL) that hospitals routinely gave to postpartum mothers contained infant formula samples and industry coupons for formula, baby food and bottles, and industry-printed guide to breastfeeding. Two types of innovative gift bags were used in this study: (1) BF-INFO gift bags containing breastfeeding information and nursing supplies and (2) PUMP gift bags containing a manual breast pump as well as breastfeeding information and nursing supplies. Innovative gift bags did not contain formula samples. Common contents selected to include in the two innovative gift bags (see Table 1) were designed to support the breastfeeding mothers. For example, we provided disposable and reusable/washable breast pads for leaking milk [20, 21], water bottle for hydration [15], sample nipple cream to ease any soreness [22–25], and a DVD showing correct latching [26, 27] in addition to breastfeeding information and resources for local lactation supports. 

### 2.1. Methods

This study was a prospective intervention study. The intervention in this study was the provision of innovative discharge gift bags for mothers when they are discharged from the hospital. A cohort of breastfeeding mothers was invited to participate in the study from three maternity hospitals in Southern New Jersey during 2009 and 2010. The eligibility criteria to participate in this study were breastfeeding mothers who spoke English or Spanish, 18 years or over in age, and delivered a full-term baby. When breastfeeding mothers volunteered to participate in the study, they were assigned to groups. For example, we recruited the cohort for COMMERCIAL first and then the cohort for BF-INFO followed by the cohort for PUMP. This recruitment and group assignment procedure prevented the potential contamination between groups. The recruitment period was approximately two to three months for each cohort. Once the projected sample size for the first cohort (COMMERCIAL) was reached, we recruited the second cohort (BF-INFO) followed by the third cohort (PUMP). 

Participants received either the industry gift bag or one of the innovative discharge gift bags in the hospital. Mothers were then followed over 12 postpartum weeks to query their infant feeding methods, exclusive or partial breastfeeding, at three contact points. Mothers who were feeding the baby breast milk exclusively, without any supplementation (e.g., infant formula, other fluids, or solid food), were recorded as exclusive breastfeeding, that is, nursing at the breast or bottle-feeding with expressed milk. Mothers who supplemented their breastfeeding with infant formula or any other food/fluid were recorded as partial breastfeeding. 

On the day of the discharge (at baseline), staff nurses visited mothers to provide gift bags. The contents of the bag were reviewed with minimal commentary in a “question and answer” format. Upon receiving gift bags at baseline, participating mothers completed a survey that included demographic information and asked about their intended duration of breastfeeding, as well as obtaining contact information for followup. At 2, 4, and 12 weeks postpartum, the mothers were contacted via email/phone/postal mail to inquire about their infant feeding methods as well as their perception of the innovative gift bags. Inquiry followed a structured questionnaire composed of multiple-choice and open-ended questions. Questions included the method of infant feeding and reason for stopping exclusive breastfeeding. Participants provided free responses on their perception of the innovative gift bags. The Institute of Review Board at the university and the three maternity hospitals approved the study protocol.

### 2.2. Data Analysis

The mean duration of exclusive or partial breastfeeding was compared between groups using the analysis of variance. The Tukey's HSD test was performed to detect which specific means were significantly different from one another. The proportion of mothers who maintained exclusive or partial breastfeeding through 2, 4, or 12 weeks was compared between groups at each point using the Chi-square analysis. The Type I error rate was set at 0.05 for all statistical tests. Comments on innovative gift bags were summarized according to common concepts and frequencies. A descriptive analysis summarized the demographic characteristics of participants. Demographic characteristics were then compared between groups to test the equivalency of intervention and control groups. For demographic characteristics (i.e., marital status) that are significantly different (*P* < 0.05), an additional comparison of the mean duration (exclusive and partial) was performed between marital statuses by study group to determine the effects of intervention. Data were analyzed using Statistical Package for the Social Sciences (SPSS) version 17.0 (SPSS, Inc., Chicago, IL, USA). 

## 3. Results and Discussion

A total of 704 breastfeeding mothers enrolled at baseline across the study groups, and 386 mothers completed the 3-month duration of the study (54.8% completion rate): COMMERCIAL (*n* = 138, 61.6%), BF-INFO (*n* = 121, 46.2%), and PUMP (*n* = 127, 58.2%). The completion rate was moderate, and the final sample size was sufficient given the power of 0.8, type 1 error rate at 0.05. As shown in Table 2, most demographic characteristics, for example, mean age, degree of ethnic diversity, education levels, and socioeconomic status, were similar between groups (*P* > 0.05). However, the marital status was statistically different between groups (*P* = 0.04). A higher proportion of mothers in COMMERCIAL group were single, compared to BF-INFO and PUMP groups: 16.1% versus 6.6% versus 9.4%, respectively. Therefore, additional comparison of the mean duration of exclusive breastfeeding was performed to determine the relationship between the marital status and exclusive breastfeeding. No relationship was detected for other demographic characteristics and exclusive breastfeeding. 

Participant loss in followups (nonrespondents) was largely due to their change of contact information or absence of response to phone messages/mails. The demography of non-respondents compared to respondents who completed the study was different (*P* < 0.001). Non-respondents (versus respondents) were more likely to be younger (30.5 ± 5.7 versus 32.1 ± 4.3), single (94.2% versus 5.8%), WIC-eligible (80.9% versus 19.1%), Black (67.6% versus 32.4%) or Latina (61.9% versus 38.1%), and had fewer years of formal education (high school or under, 69.1% versus 31.7%).

The comparison of the mean duration of breastfeeding by three postpartum months demonstrated that mothers in the PUMP group maintained both exclusive and partial breastfeeding for the longest period compared to mothers in the other two groups (Table 3). There was a significantly longer duration of exclusive breastfeeding in BF-INFO (*P* = 0.01) and PUMP (*P* = 0.001) compared to COMMERCIAL, but the duration of exclusive breastfeeding between BF-INFO and PUMP was not significantly different (*P* = 0.772). Similarly, the duration of partial breastfeeding was significantly longer among mothers in PUMP than mothers in COMMERCIAL (*P* < 0.001); yet the rate was not significantly different between PUMP and BF-INFO (*P* = 0.643). The proportion of mothers (62.2%) who maintained exclusive breastfeeding through 12 postpartum weeks was significantly high (*P* < 0.001) for mothers in the PUMP group.

**Figure 1:**
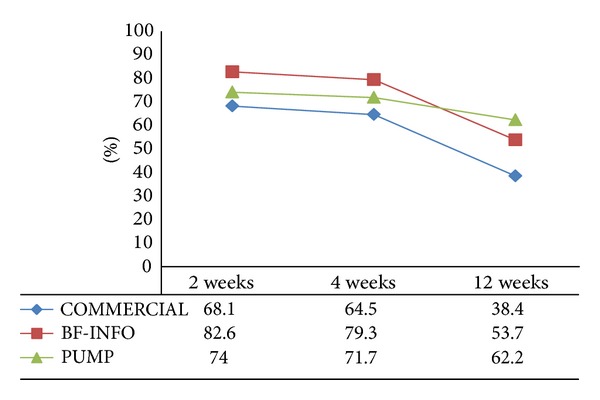
Exclusive breastfeeding rate (%) trends through 12 weeks. *P* values for comparisons between groups: at 2 weeks, *P* = 0.02, and at 4 weeks *P* = 0.03, at 12 weeks *P* < 0.001.

By 12 postpartum weeks, the rate of exclusive breastfeeding declined for all groups. However, the attrition rate of exclusive breastfeeding in PUMP group was significantly low (*P* < 0.001) compared to COMMERCIAL and BF-INFO groups: attrition rate of 11.8% versus 29.7% versus 28.9% (*P* < 0.001), respectively (see Figure 1). The rate of partial breastfeeding at 12 weeks was similar in all groups (24.6% versus 28.9% versus 26.0% = COMMERCIAL versus BF-INFO versus PUMP, *P* = 0.73). 

Marital status had a significant influence on the exclusive breastfeeding duration among mothers in BF-INFO group (*P* = 0.04). As shown in Table 4, married mothers were able to maintain the exclusivity of breastfeeding longer than single mothers: 8.09 ± 4.57 weeks versus 4.75 ± 4.62 weeks (*P* = 0.04), respectively. Mothers in PUMP group sustained exclusive breastfeeding the longest regardless of their marital status. It appears that contents of the PUMP gift bag may have influenced the exclusivity of breastfeeding for both married and single women in this study.

 Mothers described the usefulness of the innovative bags during follow-up contacts. A small proportion of mothers (3%) stated that they would have liked the electric breast pump, but the overwhelming majority of mothers (97%) appreciated the manual pump included in the bag. Some of the ways they used the manual pump were “to express a little to decrease the flow in the beginning,” to use temporarily during “emergencies such as engorgement, chapped/cracked nipples,” or to pump and store breast milk so that “husband can feed and bond with the baby.” A few mothers stated that not having the formula easily available helped her “not give in” to formula feeding. In addition, a number of mothers pointed out that latching was the most difficult part of learning how to breastfeed. The latching DVD may have facilitated their learning process because they were able to watch it “over and over” until they could successfully maintain a good latch.

## 4. Discussion 

From the findings of this study, we believe that providing discharge gift bags that contain manual pumps has a positive impact on the duration and exclusivity of breastfeeding. The exclusivity declined for all the study groups over the 12-week period, but mothers in the PUMP group sustained exclusivity most consistently through three months. Mothers in BF-INFO started with a higher rate of exclusive breastfeeding at 2 and 4 weeks, but the rate fell significantly to a level lower than mothers in PUMP group by 12 weeks. We hypothesize that mothers may be in need of technical support to sustain exclusivity for a longer duration. 

The decline of exclusive breastfeeding over three months in this study was commonly observed in other studies [28–30]. Recent data indicate that 35.0% of infants born in 2008 were breastfed exclusively through three months, falling short of the target rate of 46.2% under the *Healthy People 2020* objectives [6]. The rate of exclusive breastfeeding through three months among mothers who received the innovative PUMP bag in this study was impressive: 62.2%, well above the *Healthy People 2020* target rate. 

Adaptation to breastfeeding in early postpartum is crucial to sustain breastfeeding and its exclusivity. Mothers in this study reported that using the available manual pump eased their engorgement and initial discomfort associated with frequent breastfeeding in the early stages of breastfeeding. They were able to resolve frequently mentioned breastfeeding challenges such as insufficient milk supply, soreness/discomfort in nursing, low self-efficacy, and being tied down [31–33]. Use of the manual breast pump seemed to have facilitated the mother-infant feeding dyad. 

Various studies provide further evidence that commercial discharge bags provided by formula companies have a negative effect on the duration and exclusivity of breastfeeding [12, 13, 16–18]. In spite of this, previous studies that specifically tested the effect of gift bags free of infant formula reported conflicting results on the duration of breastfeeding [34–36]. Dungy et al. [34] tested the effect of varying content in discharge packages on the duration of exclusive breastfeeding: manual breast pump versus infant formula plus manual breast pump versus infant formula. During a 16-week postpartum followup, they found no significant difference (*P* > 0.05) in breastfeeding duration and exclusivity between groups. On the other hand, Bliss et al. [36] reported that receiving a manual pump in the discharge bags helped mothers breastfeed exclusively during early postpartum period, at 6 weeks. 

The current study provides the most recent evidence of the positive impact of using discharge bags that contain breastfeeding information plus a manual breast pump on the duration and exclusivity of breastfeeding. The role of the manual pump in early postpartum is made clearer from the mothers' descriptions of their use in this study. It is important to remember that previous studies that tested the content of hospital gift bags were conducted more than a decade ago. The positive impact of receiving the gift bag containing a manual pump in addition to breastfeeding information in the current study could be a combination of evolution in attitudes toward breastfeeding, staff support during hospital stay, and the technological advancement of the manual pump over the past decade. 

Married mothers in the current study consistently breastfed longer and maintained exclusivity more than single mothers, regardless of the type of gift bag they received. When comparing mothers in BF-INFO group, the difference of the mean exclusive breastfeeding duration in this study was large and statistically significant between marital statuses (*P* = 0.042). It may be that single mothers may lack the social support needed to utilize the breastfeeding information to its fullest, for example, unable to utilize the local lactation resources, or readily discuss with partners about lactation challenges. They may be more in need of readily available manual pumps and breastfeeding information. In addition, married mothers maybe able to stay at home with their babies and breastfeed while single mothers are more likely to need to go to work as the sole household provider. 

When comparing mothers in PUMP group, the difference of the mean duration of exclusive breastfeeding between marital statuses was minor and statistically insignificant (*P* = 0.653). This supports the potential role of the manual pump in enabling single mothers to maintain breastfeeding exclusivity. 

Limitations of this study include the lack of diversity in participants, for example, non-WIC participants, primarily white, and relatively highly educated women participated in the study. Future studies with more diverse and vulnerable populations such as WIC eligible women and working mothers can provide further understanding of needs for exclusive breastfeeding. Other limitations include having a long gap between follow-up contacts (2nd and 3rd) and relying on mother's report on infant feeding practice. More frequent follow-up contacts (e.g., every 2 weeks) could improve the completion rate. Future studies could incorporate additional methods (e.g., review of medical chart) to determine infant feeding practice to cross-validate mothers' reports. Moreover, studies that examine the rate of exclusive breastfeeding through 6 months are needed to confirm the findings of the current study. 

## 5. Conclusion

One of the most noteworthy findings from the current study is that the rate of exclusive breastfeeding for mothers provided with a manual pump was far above the rate in previous studies: 62.2% versus less than 30% [19] at 12 weeks. These results will serve as a strong motivation to shape hospital policies and practices. Hospitals that continue to provide commercial gift bags while also supporting breastfeeding during mothers' hospital stay are sending confusing signals to breastfeeding mothers. By providing breastfeeding-friendly innovative gift bags, hospitals can practice a uniform promotion strategy for breastfeeding and encourage mothers to breastfeed their babies. 

Recent studies have shown that hospitals have become more open to distributing alternative gift bags for ethical or health-based reasons [15]. These alternative bags commonly contain breast pads, baby blankets, and water bottles for the mother [15]. Adding a manual breast pump in the alternative gift bags is worth consideration for which we could provide mothers with environmental as well as technical and practical support. Hospitals, government institutions, and private industries should work together to support strategies and practices that reinforce our national effort to promote exclusive breastfeeding. 

## Figures and Tables

**Table 1:** Contents of gift bags in the study.

Gift bags	Contents
COMMERCIAL	Infant formula samples Industry coupons for infant formula, baby food and bottles Industry-printed guide to breastfeeding

BF-INFO	*Breastfeeding information* (i) How to latch and hold the baby (ii) Milk production information (iii) Sore nipples and engorgement (iv) Storing and handling of breast milk (v) Diet guide for breastfeeding (vi) A log to record daily breastfeeding and output (wet and soiled diapers) (vii) How to express breast milk using a pump (viii) Combining working and breastfeeding *Breastfeeding resources* (i) Local breastfeeding support group contact information (ii) Hospital lactation hot-lines *Breastfeeding-friendly items* (i) Nipple cream (ii) Disposable and reusable/washable nursing pads (iii) DVD on correct latching (iv) Memory bracelet (help remembering the previous nursing side) (v) Water bottle

PUMP	The same content as BF-INFO bags plus a manual breast pump

**Table 2:** Demographic characteristics.

Characteristics	COMMERCIAL *n* = 138 (%)	BF-INFO *n* = 121 (%)	PUMP *n* = 127 (%)	*P*
Marital status				0.04
Married	83.9	93.4	90.6	
Single	16.1	6.6	9.4	
Socioeconomic status				0.11
WIC^1^-eligible	15.3	6.7	7.9	
Non-WIC	84.7	93.3	92.6	
Ethnicity				0.83
Asian	3.6	1.7	5.5	
Black	3.6	4.1	3.1	
Latino	8.8	5.0	7.1	
White	81.8	86.8	81.9	
Other	2.2	2.5	2.4	
Education				0.10
Less than high school	1.5	0.8	0.8	
High school graduate	13.9	4.2	6.3	
Some college	13.9	21.7	18.1	
College graduate	40.9	49.2	42.5	
After college	29.9	24.2	32.3	
Mother's age (years) (mean ± SD)	31.24 ± 5.03	31.73 ± 4.91	32.43 ± 7.42	0.51
Birth weight (kg) (mean ± SD)	3.32 ± 0.68	3.48 ± 0.42	3.36 ± 0.67	0.07
Exclusive breast feeding intention (months) (mean ± SD)	4.76 ± 3.99	6.06 ± 4.18	6.87 ± 9.75	0.06

^1^WIC is the Special Supplemental Nutrition Program for Women, Infants, and Children.

**Table 3:** Mean durations of exclusive and partial breastfeeding compared (pair-wise) between study groups.

	COMMERCIAL (mean ± SD)	BF-INFO (mean ± SD)	PUMP (mean ± SD)	P
Exclusive (weeks)	6.12 ± 4.49	7.87 ± 4.63		0.010
	6.12 ± 4.49		8.28 ± 4.86	0.001
		7.87 ± 4.63	8.28 ± 4.86	0.772

Partial (weeks)	8.47 ± 4.35	10.48 ± 3.26		<0.001
	8.47 ± 4.35		10.89 ± 2.96	<0.001
		10.48 ± 3.26	10.89 ± 2.96	0.643

**Table 4:** Mean durations (weeks) of exclusive and partial breastfeeding compared between marital statuses.

	Exclusive (mean ± SD)			Partial (mean ± SD)		
	Married	Single	*P*	Married	Single	*P*
COMMERCIAL	6.31 ± 4.79	5.32 ± 4.83	0.372	8.70 ± 4.26	7.59 ± 4.62	0.27
BF-INFO	8.09 ± 4.57	4.75 ± 4.62	0.042	10.60 ± 3.15	8.75 ± 4.53	0.12
PUMP	8.35 ± 4.82	7.67 ± 5.40	0.653	11.00 ± 2.84	9.83 ± 3.95	0.19
